# Supplementary material for: Spatial diversity processing mechanism based on the distributed underwater acoustic communication system
Source: PLoS One. 2024 Jan 2;19(1):e0296117. doi: 10.1371/journal.pone.0296117 (PMC10760719; doi:10.1371/journal.pone.0296117)
Supplement: S1 Appendix — (PDF) [file pone.0296117.s002.pdf]

S1 Appendix. Comparison table of the related works.

Classification	Reference
**Distributed Antenna Systems**	Roh W et al. [1], Huang K et al. [2]<br>Zhuang H et al. [7], Gong J et al. [8]<br>Ibernon-Fernandez R et al. [9]<br>Dong Y et al. [10], Heath R et al. [11],<br>Yoshida S et al. [12]
**Underwater Wireless Sensor Networks**	Sathish K et al. [3–6]
**Spatial Diversity Equalizer for Wireless Communication**	Balaban P et al. [13], Li Y et al. [14]<br>Rao W et al. [15], Shen CY et al. [16]<br>Li X et al. [17], Cherifi A et al. [18]<br>Mehta R et al. [19]
**Spatial Diversity Equalizer for Underwater Acoustic Communication**	Chitre M et al. [20], Wen Q et al. [21]<br>Yang T et al. [22], Singer AC et al. [23]<br>Kim H et al. [24]
**Space Time Block Coded**	Alamouti SM et al. [25], Ganesan G et al. [26]<br>Jafarkhani H et al. [27], Jongren G et al. [28]<br>Roy S et al. [29], Guo H et al. [30]<br>Li B et al. [31], Sun L et al. [32]<br>Xiao L et al. [33], Goutham V et al. [34]
**BELLHOP Underwater Acoustic Channel Model**	Etter PC et al. [35], Porter MB et al. [36, 37]<br>Alexander P et al. [38], Gul S et al. [39]<br>Hovem JM et al. [40], Zhou M et al. [41]
