# Supplementary material for: Spatial diversity processing mechanism based on the distributed underwater acoustic communication system
Source: PLoS One. 2024 Jan 2;19(1):e0296117. doi: 10.1371/journal.pone.0296117 (PMC10760719; doi:10.1371/journal.pone.0296117)
Supplement: Attachment — Submitted filename: Response to Reviewers.pdf [file pone.0296117.s003.pdf]

Original Manuscript ID: PONE-D-22-31101

Original Article Title: "Distributed underwater acoustic diversity processing mechanism based on cross-media buoys network"

To: PLOS ONE Editor

Re: Response to reviewers

Dear Editor,

Thank you for allowing a resubmission of our manuscript, with an opportunity to address the reviewers' comments.

We are uploading (a) our point-by-point response to the comments (below) (Response to Reviewers), (b) an updated manuscript with yellow highlighting indicating changes (Revised Manuscript with Track Changes), and (c) a clean updated manuscript without highlights (Manuscript).

Best regards,

<Manli Zhou> et al.

Reviewer#1, Concern # 1: The way of redacting the sentence: "... systems [9]. [10]conducted a ..." in page 2/15, first paragraph is not very fortunate. It is suggested to improve the redaction and insert a space to separate words while typing. For example as can be seen in: "... [17] design rate ..." in the second paragraph on page 2/15.

Author response: We are sorry for this editing error. We have improved the redaction and inserted a space to separate words while typing.

On page 2/17, first paragraph: "... systems [9]. [10] conducted a ...".

Reviewer#1, Concern # 2: The way of redacting the sentence: "... channels.In practice, ..." in page 2/15, first paragraph is not very fortunate. It is suggested to improve the redaction and insert a space to separate words after a period.

Author response: We are sorry for this editing error. We have improved the redaction and inserted a space to separate words after a period.

On page 2/17, first paragraph: "... channels. In practice, ...".

Reviewer#1, Concern # 3: It is suggested to improve the same in: "... [13]proposed a ..."; "... [14]studies a ..."; "... [16]cast space–time codes ..."; "... [19]proposed ..."; "... advantages:The phase ..."; "... compensation.The combining ...".

Author response: We are sorry for this editing error. We have improved the redaction and inserted a space to separate words while typing. Since we have added new references, the numbers of references have also been updated.

On page 2/17, first paragraph: "To address this problem, [13] proposed a ..."; "... [14] studies a ...".

On page 2/17, second paragraph: "... [18] cast space–time codes ..."; "... The proposed transmission scheme [20] combines the ..."; "... [21] proposed ...".

On page 3/17: "... advantages: The phase ..."; "... compensation. The combining ...".

Reviewer#1, Concern # 4: In page 3/15, section "BELLHOP Gaussian beam tracking model", first paragraph, the redaction is not very fortunate: "... sound field research22 ...". It is suggested to improve the redaction.

Author response: We are sorry for this editing error. We have improved the redaction of Reference 22. Since we have added new references, the numbers of references have also been updated.

On page 4/17, first paragraph: "... sound field research [27] ...".

Reviewer#1, Concern # 5: It is suggested to improve the redaction in: "... model [24]is shown..." in page 3/15, section "BELLHOP Gaussian beam tracking model", second paragraph.

Author response: We are sorry for this editing error. We have improved the redaction and inserted a space to separate words while typing. Since we have added new references, the numbers of references have also been updated.

On page 4/17, second paragraph: "... model [29] is shown ...".

Reviewer#1, Concern # 6: In page 4/15, it is not clear if conceptually the authors are referring to h(t) as the impulse response function, defined in the equation (1), but at the same time, as h(t) to the transfer function. This could be improved.

Author response: Thank you for your valuable suggestions. I'm sorry for the conceptual confusion. If the underwater acoustic channel is regarded as a system, the input signal passes through the underwater acoustic channel to obtain the output signal. In the time domain, the impulse response is used to describe the characteristics of the system. That is, the input signal is convoluted with the impulse response to obtain the output signal of the system. In the frequency domain, the transfer function is used to describe the characteristics of the system. That is, the input signal is multiplied by the transfer function of the system to obtain the output signal of the system. In this paper, we model the underwater acoustic channel in the time domain. Therefore, h(t) should be described more accurately by impulse response.

We have corrected the transfer function h(t) to the impulse response h(t) on page 4/17, section " Underwater acoustic channel impulse response h(t)".

Reviewer#1, Concern # 7: It is suggested to improve the redaction in page 4/15: "... based on a cross-media buoys network. Based on the ...". In the same line, "based on" is used two times.

Author response: Thank you for your valuable suggestions. We have rewritten this part for better readability.

On page 5/17, first paragraph: "This paper constructs a DUA-DCS based on a cross-media distributed buoys network. Because the multiple underwater acoustic communication links distributed in a certain area have the characteristics of mutual statistical independence, the system uses the buoys network with motorized deployment to convert the ’point-to-point’ variable parameter channel into a ’point-to-multipoint’ or ’multi-point-to-point’ quasi-constant parameter stationary channel.".

Reviewer#1, Concern # 8: In equation (4), page 6/15, the parameter "e" is not defined. It is suggested to improve the redaction.

Author response: Thank you for your valuable suggestions. We have defined the parameter "e" and updated the manuscript.

On page 7/17, fourth paragraph: "$e(i)$ denotes the error between the desired output value $d(i)$ of the equalizer and the decision value $\hat{y}(i)$ at the time i."

Reviewer#1, Concern # 9: It is suggested to improve the redaction in: "... Initialization:ω (0) = ..."; "... Among them,y (n) = ..." in page 7/15, and insert spaces to separate words while typing.

Author response: We are sorry for this editing error. We have improved the redaction and inserted a space to separate words while typing.

On page 7/17: "... Initialization: ω (0) = ..."; "... Among them, y (n) = ...".

Reviewer#1, Concern # 10: In page 7/15, "... $y_b(n) = [y(n-1), \cdots, y(n-N_3)]$. ...", the parameter "$N_3$" is not defined, or the question is if it corresponds to "N_2", the order of FBF. This could be improved.

Author response: Thank you for your valuable suggestions. The parameter "N_3" corresponds to "N_2", that is, the order of FBF. We have corrected it in the manuscript.

On page 7/17: "$y_b(n) = [y(n-1), \cdots, y(n-N_2)]$".

Reviewer#1, Concern # 11: In page 8/15, the style of the equation (16) is not very lucky: "... $h_{j,i}(t) = h_{j,i}(t+T) = h_{j,i}, t = 1,2,\cdots p$ ". This one could be improved.

Author response: Thank you for your valuable suggestions. We have modified equation (16) according to the correct format.

On page 10/17, equation (16): "$h_{j,i}(t) = h_{j,i}(t+T) = h_{j,i}$

Where $t = 1,2,\cdots p$."

Reviewer#1, Concern # 12: In page 8/15, the style of the equation (18) is not very lucky: "... xi = argmind2...". This one could be improved.

Author response: Thank you for your valuable suggestions. We have modified equation (18) according to the correct format.

On page 10/17, equation (18): " $\hat{x}_i = arg \min_{\hat{x}_i \epsilon S} d^2(\tilde{x}_i, \hat{x}_i) + (\sum_{i=1}^{N_i} \sum_{j=1}^{N_t} \left|h_{j,i}\right|^2 - 1)|x_i|^2$

Where $i = 1,2,\cdots k$."

Reviewer#1, Concern # 13: In page 11/15, it is suggested to improve the redaction both in: "... Case 2:Verify ... ", and "... Case 3:This ..." Insert spaces to separate words while typing.

Author response: We are sorry for this editing error. We have improved the redaction and inserted a space to separate words while typing.

On page 12/17, second paragraph: "... Case 2: Verify ... ".

On page 12/17, fourth paragraph: "... Case 3: This ...".

Reviewer#1, Concern # 14: For consistency in writing, to refer to the equations, the authors use on page 8/15: "... It satisfies Eq (12)...". However, on page 11/15, the authors employ: "...as Eq 14 and Eq 15. ...". It is suggested to improve the redaction to be consistent in writing.

Author response: Thank you for your valuable suggestions. For consistency in writing, we have corrected "...as Eq 14 and Eq 15 ..." to be consistent with "... It satisfies Eq (12) ...".

On page 12/17, fourth paragraph: " Case 3: This simulation is to verify the influence of the transmit antennas' number on the system performance in the downlink under the same environmental parameters. The complex orthogonal transmission matrixes of two transmitting antennas and three transmitting antennas are designed as Eq (14) and Eq (15)."

Reviewer#1, Concern # 15: In page 12/15, section Lake experiments and results. It would be opportune to know if the authors before the field measurements have carried out, or not, a calibration procedure of the hydrophones and of all the experimental setup. If some calibration procedure was carried out, it would be appropriated that the authors describe it.

Author response: Thank you for your valuable suggestions. Before the field measurements, we carried out a calibration procedure of the hydrophones and all the experimental setups. We updated the manuscript by describing this part in detail.

On page 12/17: Add "Before the lake experiments, the hydrophones, and other experimental equipment have been professionally calibrated by the manufacturer. In the meantime, we have completed tasks such as testing electrical connections and ensuring communication link availability in a water tank environment. The transmitter sends a QPSK modulation signal through the transducer connected to the underwater acoustic communication apparatus module. The receiver collects underwater acoustic signals through a transducer connected to a digital collector. The obtained underwater acoustic signal is processed by Matlab. If the data can be successfully demodulated, it is proved that the communication link is passable and the electrical connection is correct."

Reviewer#1, Concern # 16: In page 12/15, it is suggested to improve the redaction in: "... Fig 15.The equipment ..." and insert space to separate words while typing.

Author response: We are sorry for this editing error. We have improved the redaction and inserted a space to separate words while typing.

On page 13/17, first paragraph: "... Fig. 15. The equipment ...".

Reviewer#1, Concern # 17: Table 3, in page 12/15: The way of presenting the results in Table 3 is not very fortunate. It is suggested to improve the presentation.

Author response: Thank you for your valuable suggestions. We modified Table 3 according to the standard of the three-line table. To make readers better understand, Table 3 is explained in detail in the manuscript. At the same time, to maintain the consistency of the format of all tables, we also modified Table 1, Table 2, and Table 4 to the format of the three-line table.

On page 13/17, first paragraph: add "On the signal transmitting ship, the personal computer (PC) is connected to the underwater acoustic communication apparatus, and then the underwater acoustic communication apparatus is connected to the transducer. On the signal-receiving ship, the PC is connected to the digital collector, and then the digital collector is connected to the transducer. Among them, YOKOGAWA SL1000, HIOKI MR6000, and Altai represent the models of digital collectors."

Reviewer#1, Concern # 18: Throughout the manuscript, each of the "Fig" abbreviations should carry a period. This is, for example, "Fig. 15". This one could be improved.

Author response: Thank you very much for your valuable comments, we have modified each of the "Fig" to "Fig." according to your comments.

Reviewer#1, Concern # 19: In the references, the authors only show one article published in 2019. No article is reviewed between the years 2020 to date. This could be improved. It is suggested that the authors update the references according to the state of the art.

Author response: Thank you for your valuable suggestion. We updated the manuscript by adding new references between the years 2020 to date.

On page 2/17, first paragraph: add [15] in " In [15], the received signal is split into two streams. One stream is equalized after time reversal (TR) combining. Another stream is flipped in the time domain, processed by TR-combining and equalization, and then flipped in the time domain again. After processing, each stream is conducted diversity combining."

On page 2/17, first paragraph: add [16] in " [16] employed spatial diversity and antenna beamforming methods to significantly improve the signal quality performance of wireless networks and enhance the reliability by decreasing the bit error probability."

On page 3/17, first paragraph: add [24] in " In [24], joint time-reversal space-time block coding and adaptive equalization filtered multitone underwater acoustic communication method was proposed. The effectiveness of the proposed method is verified by simulation analysis and real experimental data collected from an indoor pool communication trial."

On page 3/17, first paragraph: add [25] in " [25] proposed a generalized space-time block coded spatial modulation scheme for open-loop massive multiple-input and multiple-output downlink communication systems. The information bits are divided into multiple groups with each group modulated by the spatial modulation, where the SM symbols are invoked for OSTBC and quasi-orthogonal STBC structures."

On page 3/17, first paragraph: add [26] in " [26] proposed space-time block coded non-orthogonal multiple access (STBC-NOMA) for underwater acoustic sensor networks (UASNs) to improve reliability by exploiting transmit diversity and spectral efficiency. Results show that STBC-NOMA can significantly enhance the performance of UASNs without the need for prior channel state information status at the transmitter."

[15] Kim H, Choi KH, Choi JW, Bae HS. Bidirectional Equalization for Long-Range Underwater Acoustic Communication in BLAC18. In: 2019 Eleventh International Conference on Ubiquitous and Future Networks (ICUFN). IEEE; 2019. p. 52–53.

[16] Mehta R. Optimal receive beamforming in spatial antenna diversity system using evolutionary genetic algorithm. Array. 2021; 10:100053.

[24] Sun L, Yan M, Li H, Xu Y. Joint Time-Reversal Space-Time Block Coding and Adaptive Equalization for Filtered Multitone Underwater Acoustic Communications. Sensors. 2020;20(2):379.

[25] Xiao L, Chen D, Hemadeh I, Xiao P, Jiang T. Generalized space-time block coded spatial modulation for open-loop massive MIMO downlink communication systems. IEEE Transactions on Communications. 2020;68(11):6858–6871.

[26] Goutham V, Harigovindan V. Space–time block coded non-orthogonal multiple access for performance enhancement of underwater acoustic sensor networks. ICT Express. 2022;8(1):117–123.

Thanks again for your advice and I hope to learn more from you.
