# Supplementary material for: Spatial diversity processing mechanism based on the distributed underwater acoustic communication system
Source: PLoS One. 2024 Jan 2;19(1):e0296117. doi: 10.1371/journal.pone.0296117 (PMC10760719; doi:10.1371/journal.pone.0296117)
Supplement: Attachment — Submitted filename: PONE-D-22-31101R1.docx [file pone.0296117.s004.docx]

In this article, “Distributed underwater acoustic diversity processing mechanism based on cross media buoys network”. The author must improve the manuscript. However, I have some comments and suggestions for the authors as follows:
1. It is better to revise or suggest another appropriate title for this Manuscript to reflect the details of the work.
2. The abstract does not communicate effectively; it should be changed.
3. The paper organization should be revised with more details about the proposed work in this manuscript.
4. In particular, the paper’s contribution is unclear compared to existing works in the literature, and there lacks a necessary comparison with the existing image enhancement methods. Thus, the current version cannot be recommended for acceptance.
5. Please add a list of Abbreviations (Acronym) after Conclusion Section,
6. In the introduction, the authors did not give clearly describe the new methods and contributions that lead to improving the comprehensive survey of image enhancement methods compared to existing works. Please explain the new comprehensive contributions of this work (methodology or algorithm?) that will improve the image enhancement existing works more specifically.
7. The authors mentioned only some related work, but without enough details. So it is necessary to add existing works in the related work section with the comparison table to improve this manuscript. Please revise it.
8. There are too many spelling and grammar mistakes in the paper. It needs proper spelling and grammar checking.
9. Most mentioned Figures includes small details; so they are not clear for the readers to distinguish the difference and the performance improvement of image enhancement methods. Add full description for all the results.
10. In conclusion, you can show briefly the percentage of improvement of image enhancement methods interms of quantitative evaluation measures.
11. What are the trends and future direction of applying those proposed method? Please give your suggestions.
12. Add some recently papers (2020-2023) in the manuscript.

https://www.mdpi.com/1424-8220/23/10/4844
https://doi.org/10.3390/electronics12061287
https://www.mdpi.com/2224-2708/11/4/64
https://www.hindawi.com/journals/jcnc/2022/9418392
https://doi.org/10.3390/jsan11040064
